# Evaluation of an ultrasound-guided freeze-core biopsy system for canine and feline brain tumors

**DOI:** 10.3389/fvets.2024.1284097

**Published:** 2024-04-09

**Authors:** Brian S. Adams, Dominic J. Marino, Catherine A. Loughin, Leonard J. Marino, Teresa Southard, Martin L. Lesser, Meredith Akerman, Patrick Roynard

**Affiliations:** ^1^Department of Surgery, Long Island Veterinary Specialists, Plainview, NY, United States; ^2^The Section of Anatomic Pathology, Department of Biomedical Sciences, Cornell University, Ithaca, NY, United States; ^3^Biostatistics Unit, North Shore—LIJ Health System Feinstein Institute for Medical Research, Manhasset, NY, United States; ^4^Veterinary Medical Center, The Department of Neurology and Neurosurgery, Ohio State University, Columbus, OH, United States

**Keywords:** brain tumor, MRI, veterinary, ultrasound-guided, freeze-core biopsy

## Abstract

**Objective:**

To determine if a single brain biopsy utilizing a freeze-core needle harvest system Cassi II under ultrasound guidance provides a diagnostic sample; to evaluate the technique's efficacy in procuring diagnostic samples in comparison with “open” surgical biopsies; and to describe intraoperative complications associated with the technique.

**Study design:**

Experimental clinical study.

**Animals:**

Seventeen dogs and four cats with magnetic resonance imaging (MRI) diagnoses of readily surgically accessible intracranial masses.

**Methods:**

Immediately prior to surgical biopsy (SB), freeze-core biopsy (FCB) sample was obtained from each patient under ultrasound guidance.

**Results:**

Histopathology results from single FCB samples were found to be in 100% agreement with the SB samples. Freezing artifact was minimal and did not interfere with histopathologic interpretation. There were no intraoperative complications specifically attributable to the use of the FCB system.

**Conclusion:**

Based on the results of this small experimental study, the FCB system is expected to safely yield diagnostic quality intracranial masses biopsy specimens.

**Clinical significance:**

This system has the potential of obtaining diagnostic biopsies of more deeply seated brain lesions (i.e., intra-axial tumors considered inaccessible or with large risks/difficulties by standard surgical means) which would provide a definitive diagnosis to guide appropriate therapy.

## Introduction

In patients with suspected intracranial disease, it is possible to provide a presumptive neuroanatomic localization based on history, signalment, and complete neurologic examination. Cross-sectional advanced imaging (e.g., computed tomography [CT] or magnetic resonance imaging [MRI]) is needed to confirm and accurately delineate the localization (1, 2). Although these advanced imaging modalities have greatly improved the ability to treat small animal intracranial diseases (neoplastic, inflammatory, and cerebrovascular brain lesions), they are sufficiently unreliable to provide a definitive diagnosis (3, 4), but the combined examination of Cerebrospinal fluid (CSF) analysis may help rule out inflammatory processes and support a diagnosis of neoplasia (5). Although both CT and MRI provide valuable detail regarding the presence, location, and size of intracranial masses, along with effect on adjacent intracranial structures (e.g., ventricular system), the superior detail afforded by MRI makes it the preferred choice for imaging the brain (6). Several studies have shown that MRI can render a presumptive diagnosis for some canine brain tumors; however, as in human patients, the accuracy varies substantially (3, 4, 7–14).

Histopathologic evaluation remains the “gold standard” for making a definitive diagnosis with an intracranial lesion and is often a necessary component for accurate treatment planning (12–15). Unfortunately, permission to biopsy is not always granted by owners because of financial limitations and the risk of morbidity. Various brain biopsy techniques in conjunction with imaging have been described in human and veterinary patients. These include free-hand vs. stereotactic systems, frame-based vs. frameless systems, and the use of various imaging modalities such as ultrasound-guided, CT-guided (12, 16–23) and MRI-guided (21, 24–26). With each of the aforementioned techniques, the current recommendation is to harvest multiple tissue samples to improve success; however, this may result in increased morbidity (21, 24, 27–29). Morbidity and mortality associated with each of the aforementioned imaging-assisted biopsy methods are difficult to compare since some dogs also had surgical removal of the brain mass immediately following the biopsy procedure. Moissonnier (19) reported a 27% morbidity and an 8% mortality, while Koblick (16) reported 12% morbidity and 7% mortality.

One theoretic advantage of the freeze-core biopsy (FCB) technique is that a single biopsy sample would yield diagnostic results owing to the enhanced tissue adherence of the tumor to the frozen sampling needle, thus lowering the risk of patient morbidity (28, 29). The main indication for the use of a small biopsy instrument with ultrasound guidance for brain tumors is for lesions that are not readily accessible surgically (e.g., deep-seated tumors). These less accessible lesions are often not biopsied due to fear of unacceptable morbidity and mortality associated with conventional surgical biopsy (SB). To validate both the new proposed biopsy technique and the FCB biopsy instrument, the authors conducted this initial study on readily accessible intracranial masses so that the biopsy material attained via conventional SB could be used as a comparison or “gold standard.” This study describes a new biopsy technique with ultrasound guidance of a FCB system, Cassi II^1^, to obtain samples of brain tumors, along with any immediate intraoperative complications. We hypothesized that the technique would provide diagnostic samples and that histological diagnosis would be similar to that obtained with SB.

## Materials and methods

### Patient inclusion and data collection

Dogs and cats with an MRI diagnosis of an intracranial mass treated with craniotomy and mass removal at our hospital over three consecutive years were admitted into the study. Only patients with masses that were considered readily accessible via a standard transfrontal or standard/modified lateral (rostrotentorial) craniectomy were included. The following information was recorded: signalment, bloodwork (e.g., complete blood count and serum biochemistry profile), neurologic examination findings, MRI results, histopathology from the 10g Cassi II freeze-core biopsy (FCB) and from surgical biopsy (SB), intra-operative complications, post-operative complications and pre-and post-operative medical therapy.

### Anesthesia and pre-operative preparation

Each patient was anesthetized for surgery in a similar manner. Patients were pre-medicated with atropine (0.022–0.044 mg/kg subcutaneously), Maropitant 1 mg/kg IV and hydromorphone (0.1 mg/kg subcutaneously) and induced with propofol (3–6 mg/kg IV). Anesthesia was maintained with isoflurane. Intravenous cefazolin was administered (22 mg/kg) at the beginning of surgery and every 90 min during surgery. Mannitol (0.5 g/kg IV over 10–15 min) and methylprednisolone sodium succinate (30 mg/kg IV) were administered pre-operatively. The MRI scans were performed using a 3.0-Tesla scanner^2^,^2^ within 7 days prior to surgery in all patients. Sagittal and transverse T2-weighted images (fast spin echo), sagittal and transverse T1-weighted images with and without the IV administration of gadolinium (Magnevist^®^, gadopentate dimeglumine, Bayer Healthcare Pharmaceuticals; 0.1 mmol/kg) were obtained. The patient's head was clipped from the level of C1 to approximately the level of the infraorbital foramina. The animal was then placed in sternal recumbency with the head slightly elevated and head and neck at an ~90° angle to each other and prepared aseptically for surgery. Care was taken not to compress the external jugular veins.

### The Cassi II freeze-core biopsy system instrumentation

**Figure 1:**
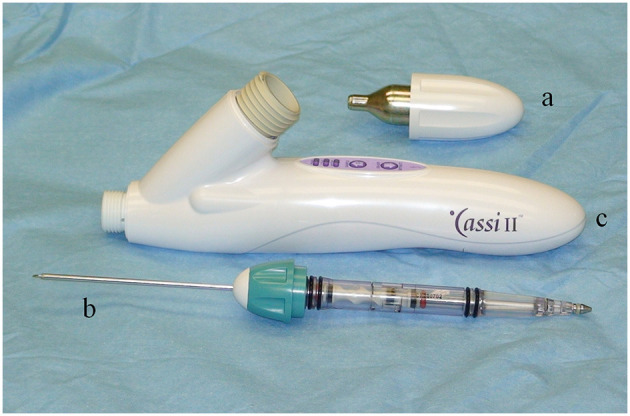
The Sanarus Cassi II freeze-core biopsy system utilizes a CO_2_ cartridge **(a)** to provide cooling for the quick “stick freeze” and a combined securing needle and a rotating cutting cannula **(b)**. The hand piece contains the operational control buttons **(c)**.

**Figure 2:**
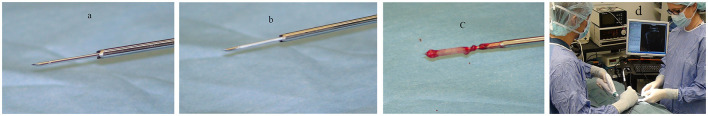
The securing needle **(a)** stabilizes the specimen using contact freezing **(b)** while the rotating cutting cannula advances and retracts revealing the biopsy specimen **(c)**. Typical surgical set-up and positioning for biopsy **(d)**.

The Cassi II freeze-core biopsy system (Figure 1) uses an ultrasound-guided, 19-gauge x 2 cm length securing needle and a 10-gauge serrated cutting cannula. A CO_2_ quick “stick freeze” localizes the target tissue around the securing needle, followed by advancement of the rotating cutting cannula. The securing needle stabilizes the specimen using contact freezing while the rotating cutting cannula minimizes distal displacement of the biopsy specimen during harvest resulting in the frozen core sample (Figure 2). The biopsy specimen obtained is ~1.5–2 cm long and 3–4 mm wide.

### Surgical technique

**Figure 3:**
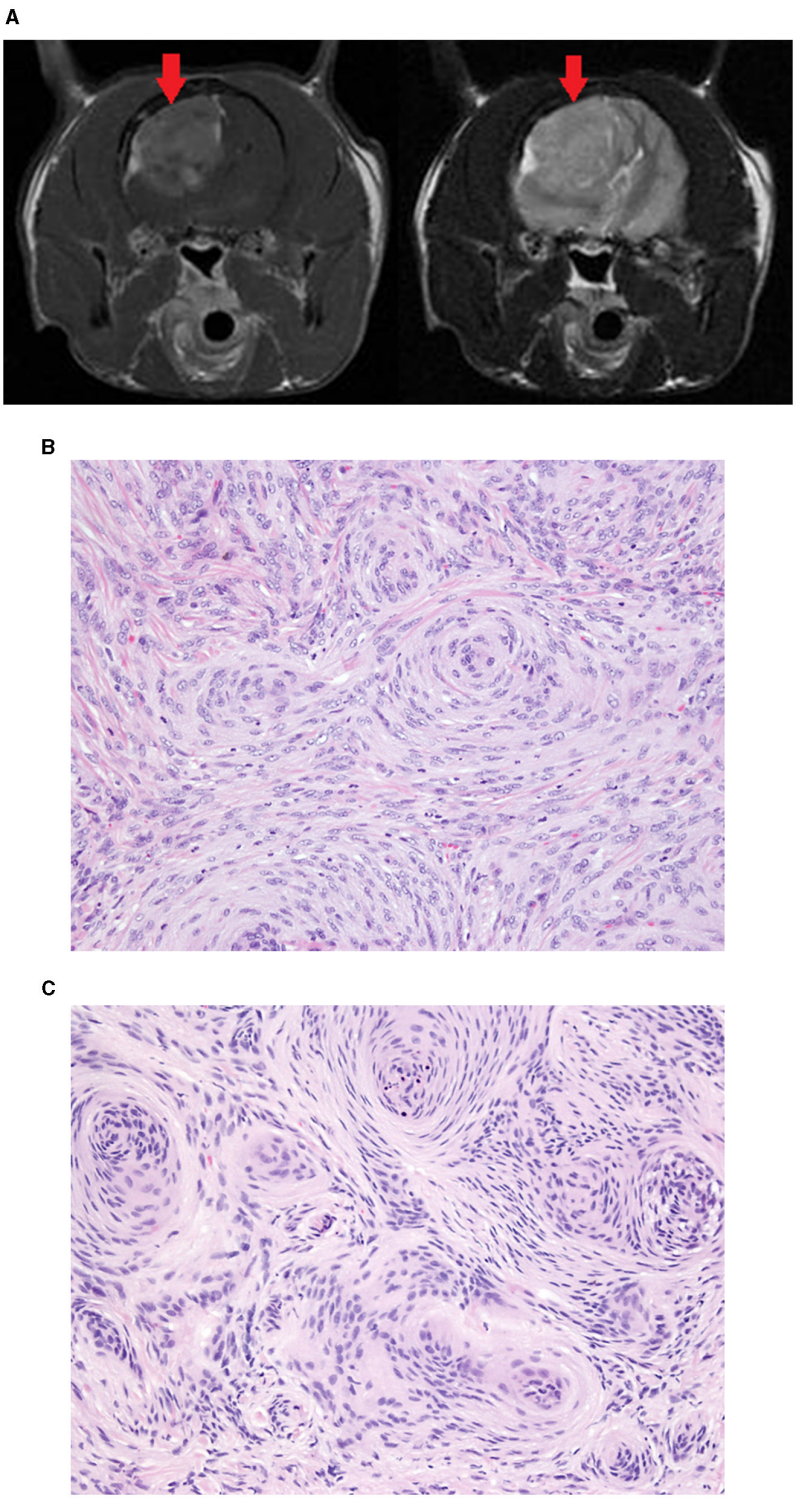
Meningioma removed from the parietal lobe of a 9-year-old DSH. Transverse T1W post-contrast (left) and T2W (right) MR images **(A)** showing the large mass (red arrow). In both the surgical biopsy **(B)** and the Cassi biopsy **(C)** the mass was diagnosed as a meningioma. The Cassi biopsy samples have shrunken nuclei with darker staining chromatin and mild loss of nuclear and cytoplasmic detail compared with surgical biopsy samples.

The surgical procedure was performed on all patients by the same primary surgeon (DJM). Either a transfrontal or a lateral rostrotentorial craniectomy was performed and access to the appropriate region of the brain based on MRI findings as previously described (1, 21, 30, 31). The craniectomy was widened using a rongeur to allow for the ultrasound probe footprint (1.75 cm) as well as an additional 0.5 cm “working space” to accommodate the FCB instrumentation. A dedicated intraoperative ultrasound system, Afia, E-Technologies^3^, which includes sterilized 12 and 20 MHz microconvex probes was utilized for the FCB technique. After successful identification of the mass with intraoperative ultrasonography, the underlying meninges were incised. Care was taken to avoid lacerating the dorsal sagittal sinus within the falx cerebri. Bleeding was minimal and was controlled with bipolar cautery and Gel Foam (Baxter Healthcare Corp, Hayward CA) sponge. The FCB stabilization needle was placed intralesionally using intraoperative ultrasonography (see text footnote 3), the freeze-core biopsy system activated, the instrument rotated 90° and withdrawn. One FCB biopsy was harvested for each tumor (Figure 3). Mass removal was then achieved as previously described under 3X magnification using an expanded field telescope^4^. All visible tumor and tissue suspected of being abnormal based on the MRI were removed via manual manipulation and aspiration, and the empty cavity flushed with saline (31, 32). No attempt to fully close the dural defect was made, and the craniectomy site was covered with hemostatic material (Gelfoam). The bone plate was not replaced. The temporalis muscle and subcutaneous tissues were apposed with simple interrupted sutures using *3-0* or *4-0* polydioxanone (PDS) suture material. Biopsy specimens were labeled according to method of harvest, SB vs. FCB, and submitted for histopathologic analysis.

### Histopathology analysis

Both SB and FCB tissue samples were fixed in 10% buffered formalin, processed routinely, and stained with hematoxylin and eosin. In the assessment of the histology samples, the evaluator was blinded to the patient origin of the SB and FCB tissue samples. Diagnosis was based on histologic examination of tissues. A published scoring system was unable to be used because several tumor types were assessed. Each biopsy was evaluated on four components: (1) mitotic rate, (2) cellular atypia, (3) necrosis, and (4) inflammation. A scoring system was used to assess each of the components on a scale of 1–3. For mitotic rate: 1 = < 2 mitoses/10 high power fields (HPF), 2 = 3–10 mitoses/10 HPF, and 3 = >10 mitoses/10 HPF. For necrosis, cellular atypia, and inflammation the scoring was as follows: 1 = mild, 2 = moderate, and 3 = severe.

### Statistical methods

Descriptive statistics were calculated for the *n* = 21 animals. Frequencies and percentages were computed for categorical data and mean ± standard deviation; median, minimum, and maximum were computed for continuous data. For continuous outcomes (i.e., mitotic rate and inflammation score), the method of Bland and Altman was used to examine correlations between the pairs, FCB and SB (33). This method utilizes analysis of covariance techniques to calculate correlations between pairs of variables collected as repeated measurements, i.e., if the FCB Mitotic rate is correlated with the SB Mitotic rate. Bland-Altman plots were constructed but did not influence the analysis in any way and were therefore, not reported. The 95% “limits of agreement” were calculated such that if both the lower and upper limits are small (in absolute value), then the two measurements can be considered equivalent. On the other hand, if either of the limits is large, then the two measurements are not equivalent. Whether a limit is considered small or large depends, in part, on clinical judgment and on the size of the limit relative to the structure or, in this case, distance, being measured.

In a similar way, the kappa (κ) coefficient was used as the measure of agreement between the FCB and SB separately for categorical outcomes such as mitotic score, Cellular atypia, Necrosis, and Inflammation. The corresponding 95% confidence interval for each of these kappas was calculated and interpreted in a way analogous to that of the Bland-Altman limits of agreement. That is, if the lower limit of the kappa confidence interval was unacceptably low then this was sufficient grounds for stating that agreement had not been established. The following guidelines outlined by Landis and Koch (34–36) were used to characterize the strength of agreement for the kappa coefficient: ≤ 0.20 = poor, 0.21–0.40 = fair, 0.41–0.60 = moderate, 0.61–0.80 = good, and 0.81–1 = very good (34–36). These descriptors also were used for describing the lower 95% confidence interval.

### Post-operative care and follow-up

After recovery, patients were monitored in intensive care for 48 h and were administered intravenous (IV) isotonic fluids (0.45%NaCl/2.5% dextrose, 66mL/kg/day) supplemented with 10 mEq potassium chloride/500 mL, until each patient was drinking and eating on their own. No intracranial pressure monitoring system was used in this study. Recovery time was monitored prospectively, but could not be retrieved accurately at the time of writing of this manuscript (due to a change in hospital software).

Postoperatively, IV buprenorphine (0.3 ug/kg, q 8 h) and IV cephalexin (22 mg/kg, q 8 h) were administered for 24 h. Temperature was monitored hourly until normal for 3 or more readings. Electrolyte analyses were performed at the surgeon's discretion. Methylprednisolone sodium succinate (30 mg/kg IV) was administered 6 h after the preoperative dose and prednisone administration was begun (0.5 mg/kg) every 12 h subcutaneously if the patient was not eating and orally when the patient was eating.

## Results

Seventeen dogs and four cats had ultrasound assisted FCB mass biopsy and subsequent SB mass removal, thus meeting the criteria for study inclusion. The 17 dogs included the following breeds: three Labrador retrievers, three Golden retrievers, and one each of Pitbull, Cocker spaniel, mixed-breed, miniature Schnauzer, Dachshund, Australian Shepherd, Shetland Sheepdog, Cockapoo, German Shepherd dog, Boston terrier, and Maltese. The four cats were domestic short hair cats. The mean age of the dogs at the time of surgery was 9.4 years (range: 4–14), while the mean age for cats was 15.3 years (range: 14–18). There were 10 male castrated, seven female spayed dogs and two male castrated, and two female spayed cats. The mean weight of the dogs was 23.2 kg (range: 8.6–36.8 kg) and 5.1 kg (range: 2.7–9.0 kg) for the cats.

Neuroanatomic localization based on MR imaging, mass dimensions, and FCB and SB histopathology results are summarized (Table 1). There were no intraoperative or post- operative deaths or complications noted.

**Table 1:** Cassi II freeze-core biopsy and surgical biopsy histopathology results, neuroanatomic localization based on MR imaging, and mass dimensions are summarized.

**Number**	**Cassi II freeze-core biopsy**	**Surgical biopsy**	**MR localization**	**Height/cm**	**Width/cm**	**Length/cm**
1	Pituitary Adenoma	Pituitary Adenoma	Midline extra-axial contrast-enhancing diencephalic mass contiguous with the pituitary gland and extending into the third ventricle.	1.8	1.5	2.3
2	Meningioma	Meningioma	Extra-axial contrast-enhancing masses in the right olfactory bulb region associated with the falx.	1.5	0.8	1.1
3	Oligodendroglioma	Oligodendroglioma	Intra-axial contrast enhancing mass on the floor of the left cranial and middle fossa extending from the caudal aspect of the olfactory bulb to the rostral aspect of the diencephalon.	1.4	1.3	2.2
4	Meningioma	Meningioma	Multi-cystic contrast-enhancing mass on either side of the rostral falx in the region of the olfactory bulb and frontal lobe regions.	2.6	1.1	2.9
5	Meningioma	Meningioma	Multilobulated mass extending from the olfactory bulb region to mid-diencephalon on the left side with patchy contrast enhancement.	2.0	1.3	2.8
6	Adenocarcinoma	Adenocarcinoma	Extra-axial olfactory bulb mass extending into the right ethmoid regions.	3.0	1.8	2.6
7	Hemangiosarcoma	Hemangiosarcoma	Intra-axial olfactory bulb mass extending into the frontal lobe, and rostral diencephalon.	1.9	1.6	4.4
8	Meningioma	Meningioma	Extra-axial mass in the dorsal left temporal and rostral occipital regions of the cerebrum extending to the level of the falx medially.	1.6	1.5	2.0
9	Meningioma	Meningioma	Extra-axial contrast-enhancing mass with medial cystic component in right cerebellar hemisphere extending to the dorsal aspect of the tentorium.	1.5	1.8	1.4
10	Ependymoma	Ependymoma	Intra-axial contrast-enhancing mass extending from the left frontal through temporal lobes with left lateral ventricle being obscured.	2.5	2.2	3.2
11	Astrocytoma	Astrocytoma	Intra-axial contrast-enhancing left-sided cerebral mass, extending from the parietal through occipital lobes.	1.4	1.2	1.5
12	Meningioma	Meningioma	Extra-axial contrast-enhancing mass associated with the right cingulate gyrus, falx and dorsal aspect of the frontal lobe.	1.1	0.8	1.2
13	Adenocarcinoma	Adenocarcinoma	Extra-axial, contrast-enhancing mass involving the right olfactory bulb and frontal lobe region.	3.1	1.8	3.2
14	Meningioma	Meningioma	Extra-axial contrast-enhancing mass in dorsolateral aspect of parietal lobe	0.6	0.7	0.8
15	Meningioma	Meningioma	Extra-axial olfactory bulb/frontal lobe mass	2.1	0.9	1.6
16	Meningioma	Meningioma	Extra-axial, contrast-enhancing mass in the olfactory bulb and right frontal lobe	1.9	1.2	2.7
17	Meningioma	Meningioma	Extra-axial contrast-enhancing mass involving the falx cerebri and the L olfactory bulb, frontal, temporal, and parietal lobes.	1.2	0.8	0.7
18	Meningioma	Meningioma	Extra-axial contrast-enhancing right sided cerebral mass from parietal to occipital lobe.	1.4	2.2	2.5
19	Meningioma	Meningioma	Extra-axial, contrast-enhancing, mass in dorsal and medial aspect of the left occipital lobe.	1.4	1.3	1.5
20	Meningioma	Meningioma	Extra-axial contrast-enhancing mass in left olfactory bulb region.	1.4	0.8	1.8
21	Meningioma	Meningioma	Extra-axial contrast enhancing mass on the dorsal aspect of the right parietal and occipital lobes.	1.3	2.1	2

A single FCB sample of an intracranial mass is of diagnostic quality. The FCB system histopathology results agreed with SB histopathology results in 100% of the 21 samples. Tissue architecture in the Cassi samples was very similar to that in the traditional biopsy samples. There were no tissue clefts, suggestive of ice crystal formation. Cells in the Cassi samples often had slightly shrunken nuclei with darker staining chromatin and mild loss of nuclear and cytoplasmic detail compared to the traditional samples. These changes were subtle and did not interfere with diagnosis. The Cassi biopsies were incisional biopsies, and therefore did not allow complete evaluation of invasiveness and surgical margins. The mitotic score agreement for the FCB and SB was very good [κ = 0.87 (95% CI: 0.61, 1.00)]. For cellular atypia, the agreement between the FCB and the SB was good [κ = 0.61 (95% CI: 0.29, 0.93)]. For cellular necrosis between the FCB and the SB, the agreement was good [κ = 0.72 (95% CI: 0.44, 1.00)] and for inflammation, the agreement for the FCB and the SB was good [κ = 0.68 (95% CI: 0.37, 0.98)]. The mitotic rate average difference was −0.89 (95% CI: −6.27, 4.48). The limits of agreement for the mitotic rate ranged from −6.27 to 4.48 on a measurement that averaged −0.89. The inflammation score average difference was −0.37 (95% CI: −1.89, 1.15). The limits of agreement for the inflammation score ranged from −1.89 to 1.15 on a measurement that averaged −0.37.

## Discussion

All the image-assisted brain biopsy techniques reported in literature and mentioned in the introduction of this manuscript utilized side-cutting aspiration biopsy needles, with a diagnostic accuracy ranging from 73 to 97% (27). Variable accuracy rates have been reported with different imaging-assisted biopsy methods in clinical cases ranging from 91 to 95% (16, 19). In the study published by Koblick (16), there was a wide variation in diagnostic yield for particular histologic subgroups, ranging from 50% for non-contrast enhancing lesions to 100% for meningiomas, however low case numbers were reported. The Cassi II freeze-core biopsy system was successful in producing brain biopsy samples that were of sufficient size and diagnostic quality to be evaluated histologically in all 21 cases (100%). This system demonstrated obtaining a diagnostic sample with a single biopsy.

Since our study was expected to include multiple tumor types, considering the reported interobserver variability in assessing brain tumor grades (e.g., gliomas) (37), and to be able to compare FCB specimen vs. SB specimen across this range of different tumors, specific tumor grades were not a criterion initially retained (criteria were mitotic rate, cellular atypia, necrosis, and inflammation). Hence, the diagnostic yield evaluated in our study was tumor type and the four criteria specified, not tumor grade. Although common to previous brain biopsy studies (16, 17), this is a limitation of our study and future studies on evaluation of FCB on deep-seated, non-readily accessible tumors should ideally include tumor grades in their report. Using our scoring system, we found very good agreement between the FCB and the SB for mitotic score, and good agreement for cellular atypia, necrosis, and inflammation [κ = 0.68 (95% CI: 0.37, 0.98)]. On histopathologic examination of the “stick-freeze” specimens, freezing artifact was minimal and did not compromise interpretation. Histopathologic results are an essential component of devising treatment plans in both human and veterinary patients with brain tumors (6, 10, 32). A surgical biopsy may not be possible for many reasons including financial, risk of morbidity, or “deep-seated” tumor locations.

MR imaging was initially responsible for the neuroanatomic localization and surgical planning, however expensive or rare equipment such as MRI or CT-guided stereotactic system were not required for the procedure. Stereotactic systems utilize three dimensional coordinates to identify biopsy targets within the brain. The use of stereotactic frames has been described in both human and veterinary patients (18–21, 25, 26). Because of the cost and technical demands of stereotactic frame systems, frameless and ultrasound assisted free-hand techniques have been developed and found to be successful in both human and veterinary medicine (38–40). Intraoperative brain ultrasound is a less expensive imaging modality, requires no sophisticated shielding for the operatory theater, offers visualization of brain tissue in real time (including perioperative changes such as brain shift and tumor size reduction) and may be more readily attainable at veterinary specialty hospitals. Ultrasonographic imaging and interpretive skills are required to successfully utilize this modality, as well as a familiarity with intracranial ultrasonographic anatomy (38). Our results are consistent with the successful findings of a previous report of an ultrasound assisted free hand brain biopsy technique (39).

The technique described herein requires two distinct skills to complete the FCB specimen harvest. Once the operative portal is created, the brain mass is imaged utilizing the intraoperative ultrasound and maintained in real time while the securing needle of the Cassi II freeze-core biopsy system is placed intralesionally. Although one surgeon can perform both tasks, it is preferable to have one surgeon or assistant generate the image while the other operates the biopsy system (see Figure 2d).

No intraoperative complications were noted with the Cassi II freeze-core biopsy system being utilized on brain tissue; hemorrhage was considered minimal.

Another limitation of our study, specifically if attempting a morbidity/mortality comparison with other biopsy techniques, is the fact that surgical removal/debulking of the tumor was performed during the same procedure, following the FCB. The craniectomies performed, having to accommodate for both ultrasound probe placement and sufficient manipulation of brain tissue to allow macroscopic debulking, were hence larger than burr hole approaches classically used for biopsy. Although this may have resulted in longer anesthetic and procedure time, subjectively large craniectomies have been advocated by experienced veterinary brain surgeons to allow better visualization, hemostasis, and overall easier surgical removal of tumors (41, 42). Intraoperative ultrasound also allows for real-time imaging without concern for intra-operative brain shift and detection of hemorrhages, possibly improving patient safety (43). Although our study did not include post-operative ICP direct monitoring nor post-operative MRI (due to cost associated), recovery in all patients was uneventful and all survived discharge from the hospital.

Based on the results of this study, the Cassi II freeze-core biopsy system safely yields diagnostic quality brain biopsy specimens. The clinical usefulness and safety of this system in obtaining biopsies of more deeply seated brain lesions (i.e., intra-axial tumors considered inaccessible or with large risks/difficulties by standard surgical means) is currently being investigated by the authors. Most of the tumors in this investigation (14/21) were meningiomas. Considering both the surgical challenges and more variable histologic nature of intracranial glial tumors, we are specifically interested in the utility of this biopsy system for the diagnosis of gliomas.

## Data availability statement

The original contributions presented in the study are included in the article/supplementary material, further inquiries can be directed to the corresponding author.

## Ethics statement

The animal studies were approved by Long Island Veterinary Specialist Ethics Committee. The studies were conducted in accordance with the local legislation and institutional requirements. Written informed consent was obtained from the owners for the participation of their animals in this study.

## Author contributions

BA: Writing—original draft. DM: Supervision, Writing—original draft. CL: Writing—original draft, Supervision. LM: Writing—original draft. TS: Writing—original draft. ML: Data curation, Writing—original draft. MA: Writing—original draft. PR: Writing—review & editing.

## Footnotes

^1^Cassi II, Scion Medical Technologies, Newton, MA 02458, USA.

^2^Achieva, Philips, Andover, MA 01810, USA.

^3^Afia, E-Technologies, Bettendorf, IA 52722, USA.

^4^Designs for Vision, Inc., Ronkonkoma, NY, USA.
